# Optimizing Biomimetic 3D Disordered Fibrous Network Structures for Lightweight, High‐Strength Materials via Deep Reinforcement Learning

**DOI:** 10.1002/advs.202413293

**Published:** 2025-01-23

**Authors:** Yunhao Yang, Runnan Bai, Wenli Gao, Leitao Cao, Jing Ren, Zhengzhong Shao, Shengjie Ling

**Affiliations:** ^1^ School of Physical Science and Technology ShanghaiTech University 393 Middle Huaxia Road Shanghai 201210 China; ^2^ State Key Laboratory of Molecular Engineering of Polymers Department of Macromolecular Science Fudan University Shanghai 200433 China; ^3^ State Key Laboratory of Advanced Medical Materials and Devices ShanghaiTech University Shanghai 201210 China; ^4^ Shanghai Clinical Research and Trial Center Shanghai 201210 China

**Keywords:** biomimetic, deep reinforcement learning, molecular dynamics simulations, network structures, stability optimization

## Abstract

3D disordered fibrous network structures (3D‐DFNS), such as cytoskeletons, collagen matrices, and spider webs, exhibit remarkable material efficiency, lightweight properties, and mechanical adaptability. Despite their widespread in nature, the integration into engineered materials is limited by the lack of study on their complex architectures. This study addresses the challenge by investigating the structure‐property relationships and stability of biomimetic 3D‐DFNS using large datasets generated through procedural modeling, coarse‐grained molecular dynamics simulations, and machine learning. Based on these datasets, a network deep reinforcement learning (N‐DRL) framework is developed to optimize its stability, effectively balancing weight reduction with the maintenance of structural integrity. The results reveal a pronounced correlation between the total fiber length in 3D‐DFNS and its mechanical properties, where longer fibers enhance stress distribution and stability. Additionally, fiber orientation is also considered as a potential factor influencing stress growth values. Furthermore, the N‐DRL model demonstrates superior performance compared to traditional approaches in optimizing network stability while minimizing mass and computational cost. Structural integrity is significantly improved through the addition of triple junctions and the reduction of higher‐order nodes. In summary, this study leverages machine learning to optimize biomimetic 3D‐DFNS, providing novel insights into the design of lightweight, high‐strength materials.

## Graphical abstract

This study investigates biomimetic 3D disordered fibrous network structures (3D‐DFNS), revealing key structure‐property relationships through modeling, simulations, and machine learning. A novel deep reinforcement learning framework optimizes network stability while minimizing weight and computational cost, offering insights into designing lightweight, high‐strength materials with enhanced structural integrity.

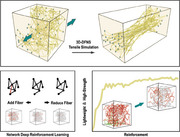

## 1 Introduction

3D disordered fibrous network structures (3D‐DFNS) are ubiquitous in nature, serving diverse mechanical functions.^[^ ^1^, ^2^, ^3^, ^4^ ^]^ For instance, the cytoskeleton maintains cellular structure,^[^ ^5^, ^6^ ^]^ collagen networks form connective tissues,^[^ ^4^, ^7^ ^]^ cocoons provide protection and spider webs facilitate hunting.^[^ ^2^, ^3^ ^]^ Compared to filling volumes with continuous materials, spanning the same space with fibrous networks is more material‐efficient and maximizes resource utilization.^[^ ^8^, ^9^ ^]^ Given that many biological structures are composed of costly proteins, minimizing material consumption is critical for the survival and efficiency of organisms.^[^ ^10^, ^11^, ^12^ ^]^ Consequently, fiber networks are commonly employed in nature to construct mechanical structures, with their performance primarily governed by their internal architecture.^[^ ^13^, ^14^ ^]^ The unoccupied volume within these fibrous networks allows fibers to move freely, change orientation, cluster, or bundle together. This flexibility in movement endows 3D‐DFNS with distinctive mechanical properties.^[^ ^15^ ^]^

The discrete nature of these 3D‐DFNS is equally important. Notably, here, the discrete nature of the 3D‐DFNS refers to its composition of individual fiber units rather than a continuous, homogeneous material. Each fiber acts as a distinct element within the network, and the specific connections and interactions among these fibers critically determine the overall properties of the material, such as mechanical strength and stress transmission capabilities.^[^ ^2^, ^16^, ^17^ ^]^ Collective deformation modes are often observed in such networks, leading to specific mechanical behaviors, such as zero‐stiffness mechanisms.^[^ ^2^, ^18^ ^]^ The multistability of these structures enables unique performance under large deformations. Additionally, fiber interactions within networks exhibit nonlocality, with mechanical responses extending over the length of network segments and involving torque transmission.^[^ ^19^, ^20^ ^]^ This is in contrast to the short‐range, strong interactions typically seen in atomic lattices. While crystalline materials exhibit high stiffness and strength, their deformation modes are restricted by lattice symmetry.^[^ ^21^, ^22^ ^]^ In contrast, fibrous network materials are softer and their anisotropy can be tuned by controlling the distribution of fiber orientations.^[^ ^23^, ^24^, ^25^ ^]^ These characteristics enable biological organisms to modulate the stiffness and strength of materials without increasing mass or volume. For example, the cytoskeleton, composed of cross‐linked protein fibers, provides structural support and its mechanical properties are essential to cellular function. The studies show that the cytoskeleton significantly stiffens under large strains, protecting cells from tensile stress.^[^ ^26^, ^27^, ^28^ ^]^ Similarly, in connective tissues, elastin and collagen fiber networks form robust mechanical systems.^[^ ^29^, ^30^ ^]^ For instance, collagen fibrils in articular cartilage provide cushioning and wear resistance,^[^ ^31^, ^32^ ^]^ while spider webs, with their 3D‐DFNS, effectively capture prey and exhibit highly tunable stress‐strain responses.^[^ ^23^ ^]^

Although the well‐recognized of advantages of 3D‐DFNS in biological systems for optimizing mechanical performance and material efficiency, their application in engineered materials remains relatively underexplored. The lightweight, tunability, and high spatial efficiency of biological 3D‐DFNS offer significant potential for structural material design, particularly in achieving efficient mechanical performance with minimal material consumption. High spatial efficiency refers to the ability to arrange structural components in a manner that maximizes mechanical performance within a confined volume, a trait characteristic prominently exhibited by biological fibrous network. In contrast, research on biomimetic material has primarily focused on ordered structures. For example, spider silk is a well‐studied biomimetic material, but most research has concentrated on the 2D orb‐web structures of orb‐weaving spiders,^[^ ^1^, ^33^, ^34^ ^]^ largely overlooking the more complex 3D‐DFNS webs woven by many other spider species.

Research on the structure‐property relationships of these 3D‐DFNS is still in its early stages, primarily due to the lack of effective experimental and simulation tools capable of deciphering their complexity. Disordered structures, compared to ordered ones, exhibit greater variability and randomness, making it challenging to infer overall performance from single‐state observations. For example, two 3D‐DFNS woven by the same spider at different times may exhibit entirely different local geometries. This high degree of variability and randomness complicate the study of such systems.^[^ ^35^ ^]^

To uncover the universal principles governing 3D‐DFNS, large datasets, and powerful analytical tools are required. In this context, deep learning techniques hold significant potential.^[^ ^36^ ^]^ For instance, Generative Adversarial Networks (GANs) can generate high‐quality 3D structural data,^[^ ^37^, ^38^ ^]^ while Long Short‐Term Memory (LSTM) networks excel at capturing complex patterns in time‐series data,^[^ ^39^ ^]^ facilitating an understanding of the dynamic evolution of 3D‐DFNS. These technologies not only assist in constructing large datasets of 3D‐DFNS but also enable the direct correlation of structure and performance through machine learning models, bypassing the challenges of complex constitutive modeling.

In this study, a constraint‐based random generation method inspired by 2D Mikado networks and 3D Voronoi meshes was developed to construct the datasets of biomimetic 3D‐DFNS. This approach enables the rapid generation of 3D‐DFNS that capture the essential features of biological 3D‐DFNS. By employing this method, thousands of biomimetic 3D‐DFNS were generated in a matter of days. These structures were then subjected to tensile simulations using coarse‐grained molecular dynamics. By combining this simulation process with batch data processing techniques, the mechanical properties of each biomimetic 3D‐DFNS were predicted within minutes, allowing the mechanical properties of thousands of structures to be predicted over several days. Analysis of the simulation results revealed that the total fiber length is the most critical factor influencing the mechanical performance of 3D‐DFNS. Given that the fibers are homogeneous, the total fiber length is directly proportional to the mass of the structure. Consequently, both the total fiber length and the mass strongly correlate with stress growth values, which are defined as the increment in stress from the initial (strain = 0%) to the final state (strain = 100%) during the stretching process. This encapsulates stress enhancement resulting from both fiber alignment and the elongation of individual fibers. Furthermore, the fiber orientation and node degree within the network exhibit weaker correlations with mechanical properties, such as stress variation. Finally, simulated experiments on the generated biomimetic 3D‐DFNS were conducted, demonstrating their robust adaptability in response to external stresses. By applying the N‐DRL algorithms, which mimic natural selection and adaptation processes, to optimize the biomimetic 3D‐DFNS inspired by natural complex 3D‐DFNS, we significantly enhanced stability while maintaining lightweight structures. This approach achieved an optimal balance between stability and mass by optimizing the trade‐off between structural weight and stability. The findings from this study are expected to provide valuable insights for the biomimetic design of lightweight, high‐strength structural materials, protective gear, impact‐resistant materials, flexible electronics, and wearable devices.

## 2 Results and Discussion

### 2.1 Construction of Biomimetic Digital 3D‐DFNS

The 3D‐DFNS data used in this study were derived from *Markus* et al.,^[^ ^40^ ^]^ who employed the Spider Web Scan (SWS) laser tomography method to capture digital 3D‐DFNS. The SWS technique utilizes a laser plane to obtain high‐resolution 2D slice images of spider webs, followed by automatic segmentation on a scanning platform. Based on these 2D images, image processing algorithms were then used to reconstruct a digital 3D fiber network.^[^ ^40^ ^]^

**Figure 1:**
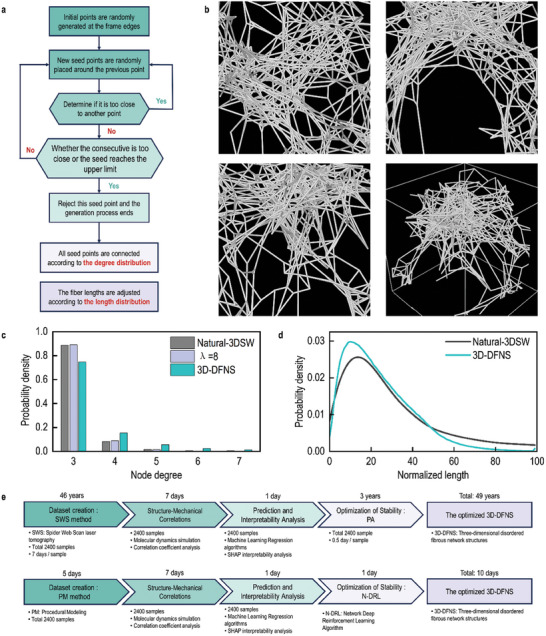
Construction of Biomimetic Digital 3D Disordered Networks. a) A comprehensive flow diagram illustrating the proposed procedural modeling methodology for generating 3D‐DFNS. b) An example of a 3D‐DFNS, encompassing the frontal, lateral, overhead and perspectives. c) A histogram of the degree distribution of fiber cross‐linking points in a 3D‐DFNS. The number of connections was regulated to align with the degree distribution of the natural 3D spider web (Natural‐3DSW), ensuring the network exhibits scaling properties typical of biological disordered networks. The overall degree distribution adhered to a power law with an exponent of 8, with triple junctions being the dominant feature, constituting 88.7% of the total connections. d) A comparison of fiber length distribution in a 3D‐DFNS and natural spider webs. The data for the Natural‐3DSW is sourced from the work of Markus et al.^[^ ^2^ ^]^ To address the inefficiency in structural generation caused by a small number of long fibers, we utilized the first 90% of the original fiber length distribution, thereby excluding the longer fibers. e) A comparative analysis of the processing time between the SWS‐PA method and the hybrid approach that integrates procedural modeling with AI analysis.

To generate a large dataset, a procedural modeling approach was adopted. As shown in **Figure** **1**a,b, a cubic box was first created, and seed points were placed using the self‐avoiding walk method, ensuring even distribution in space. These seed points were then connected and adjusted according to the characteristics of the 3D‐DFNS obtained via the SWS method, including fiber length and node degree distributions. To control fiber connections, the number of connections at each seed point was regulated to match the degree distribution, enabling the network to exhibit the scaling properties of biological 3D‐DFNS. The overall degree distribution followed a power law (*λ* = 8), with triple junctions dominating and accounting for 88.7% of the total connections (Figure 1c).

Next, the length of the fibers was adjusted to align with the expected distribution. The network was subsequently optimized using the energy minimization algorithm in the Large‐scale Atomic/Molecular Massively Parallel Simulator (LAMMPS).^[^ ^41^ ^]^ Following these adjustments, the half‐width at half‐maximum (HWHM) index for the fiber length distribution was 31.3%, which closely aligning with the biological network HWHM index of 33.1% reported by *Markus* et al.^[^ ^40^ ^]^ (Figure 1d). This procedural approach enabled us to generate large‐scale digital 3D‐DFNS that replicated the degree distribution, crosslinking density, and fiber length distribution of biological 3D‐DFNS.

Compared to the scanning‐reconstruction method for 3D‐DFNS, our strategy offers significant advantages in terms of both time and computational cost. For example, reconstructing a 3D‐DFNS with a volume of 30 cubic centimeters using the SWS method takes approximately seven days and consumes 10.644 PFLOPS (peta floating‐point operations per second) (Figure 1e). However, using the generation strategy, we could construct a network of the similar scale and complexity in mere minutes, utilizing the same computational resources—a speed improvement of several thousand times. This efficiency enables the generation of 2400 3D‐DFNS in one week, each featuring varied local microstructures but identical fiber length and node crosslinking distributions. This rapid generation capability paves the way for constructing datasets for biomimetic digital 3D‐DFNS.

### 2.2 Establishing Structure‐Property Correlations in Biomimetic Digital 3D‐DFNS

**Figure 2:**
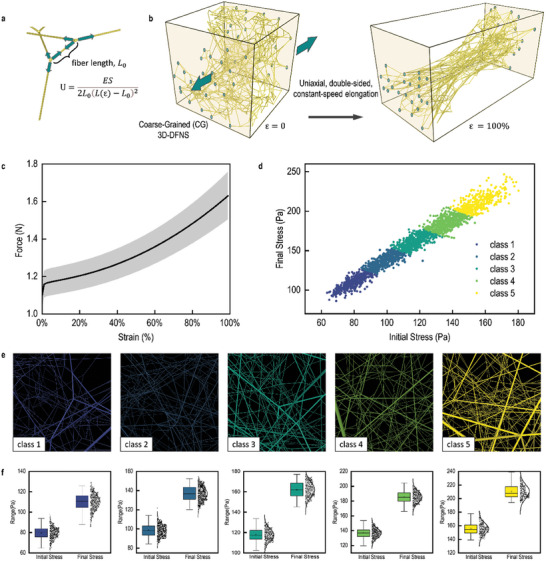
Simulation of Biomimetic Digital 3D Disordered Networks. a) A schematic representation of the forces in the coarse‐grained simulation, with harmonic forces applied exclusively at the nodes. *U* represents the harmonic potential, *E* represents the fiber modulus, *S* is the average cross‐sectional area, *L_0_* is the original fiber length and *L(ε)* is the fiber length with total strain ε. Fiber mass is concentrated at the nodes, and the mass of each crosslinking point is half the total mass of all connected fibers. b) Structural transformation of the 3D‐DFNS before and after a tensile strain of 100%. c) Tensile Force Curve from 0% to 100% Strain. All structures demonstrated the typical three‐stage force–strain behavior characteristic of biological materials. d) Classification of networks into five distinct categories using k‐means clustering, based on their initial and final stress states. e,f) Visualization of the internal structures of randomly selected networks from each class in (d). The analysis indicated that the final stress followed a broad Gaussian distribution, whereas the initial stress adhered to a narrow distribution.

The structure‐property correlations in biomimetic digital 3D‐DFNS were investigated using coarse‐grained molecular dynamics simulations. As illustrated in **Figure** **2**a, the 3D‐DFNS was simplified into a bead‐and‐stick model, where each fiber was represented by a stick connected by harmonic potentials. The harmonic bond interactions *U_b_* was calculated using the following equation:^[^ ^42^ ^]^

(1) $$\;U_{b}=\frac{ES}{2L_{0}{\left(L\left(\varepsilon \right)-L_{0}\right)}^{2}}$$

where *U_b_* represents the harmonic potential, *E* represents the fiber modulus, *S* is the average cross‐sectional area, *L*_0_ is the original fiber length, and *L*(ε) is the fiber length under total strain ε. The mass of each fiber is concentrated at the nodes, with the mass of each crosslinking point being half the total mass of all connected fibers.^[^ ^40^, ^41^ ^]^

This simplified model significantly reduced computational time. Compared to traditional bead‐spring coarse‐grained models, the bead‐and‐stick model increased simulation speed by a factor of 10 for 3D‐DFNS of comparable scale. Furthermore, the forces driving network deformation were more pronounced, making it easier to capture the structure‐property relationships under large deformations. While this model does not account for entanglement effects—since the node model only captures nonlinearity due to crosslinking—it is an effective simplification for 3D‐DFNS, where fiber entanglement is rare.

The uniaxial tensile simulation of the biomimetic digital 3D‐DFNS, applying force along a single axis, is presented in Figure 2b. At each strain step, energy minimization was applied to adjust the internal structure during stretching. Mechanical simulations were performed on 2383 sets of biomimetic digital 3D‐DFNS (with 17 groups failing during the simulation), with stress‐strain curves for all 3D‐DFNS shown in Figure 2c. All 3D‐DFNS exhibited the typical three‐stage stress‐strain behavior characteristic of biological materials. In the initial stage (strain < 20%), stress increased rapidly with strain, indicating a primarily entropy‐driven response. Simulation snapshots revealed that, despite a mere 20% strain, significant structural stretching occurred, with fibers aligning along the tensile direction. Fiber orientation increased from 33% to 42%, highlighting the load‐bearing structural elements.

In the intermediate strain range (20%‐90%), stress showed quadratic growth with strain, indicating an enthalpy‐driven mechanical response. At strains beyond 90%, the curve became linear, with the tensile modulus plateauing as most load‐bearing fibers had aligned nearly parallel to the tensile direction (fiber orientation increased from 41.7% at 20% strain to 64.9% at 90% strain). During this stage, necking was observed, and Poisson's ratio decreased from 0.575 to 0.407.

Using k‐means clustering, the 3D‐DFNS were classified into five categories based on their initial and final stress states (Figure 2d), and the internal structures of randomly selected 3D‐DFNS from each class were visualized (Figure 2e). Figure 2f shows that the stress variations before and after stretching for the five 3D‐DFNS categories followed a normal distribution, reflecting the inherent randomness of biological 3D‐DFNS. Additionally, the distribution of post‐stretching modulus significantly broadened, indicating that initial structure greatly influences mechanical properties.

**Figure 3:**
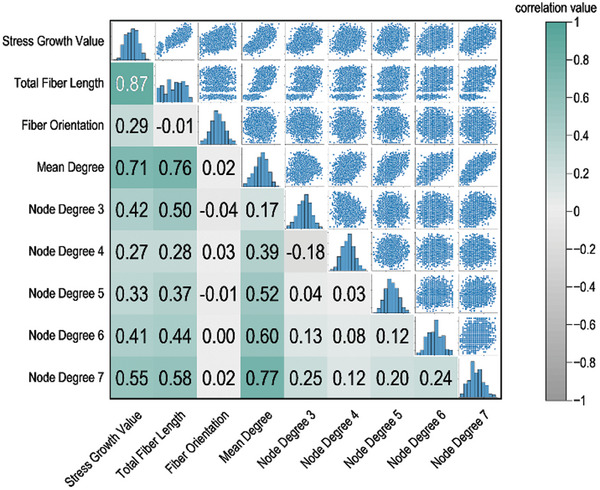
Establishing Structure‐Mechanical Correlations in Biomimetic 3D Digital Disordered Networks. Correlation matrix and scatter plot of structural features and stress growth values in 3D disordered networks.

To further analyze the relationship between structural features and mechanical properties in 3D‐DFNS, the correlation between various graph structural features (e.g., total fiber length, node degree distribution, fiber orientation) and mechanical property (stress growth values) was investigated. Fiber orientation within the 3D‐DFNS was quantified by representing the direction of each fiber as a unit vector and assessing their alignment relative to the tensile direction (x‐axis). Structural features such as total fiber length, fiber orientation, and node degree were extracted from the 2383 3D‐DFNS, while stress growth values from the tensile simulations were used as the mechanical feature. The Pearson correlation coefficients(R) between these variables were calculated and a correlation matrix heatmap (lower left part of **Figure** **3**) was constructed. The heatmap indicates that stress growth values exhibit the strongest correlation with total fiber length, with a correlation coefficient of 0.87. In contrast, fiber orientation along the tensile axis shows the weakest correlation with other structural features, with a correlation coefficient below 0.1. The scatter matrix plot (upper right part of Figure 3) further illustrates the relationships between 3D‐DFNS characteristics and stress growth values, emphasizing that total fiber length, in particular, is a critical feature with a pronounced effect on the mechanical properties of the network.

### 2.3 Evaluation of Structural Stability in Biomimetic Digital 3D‐DFNS

**Figure 4:**
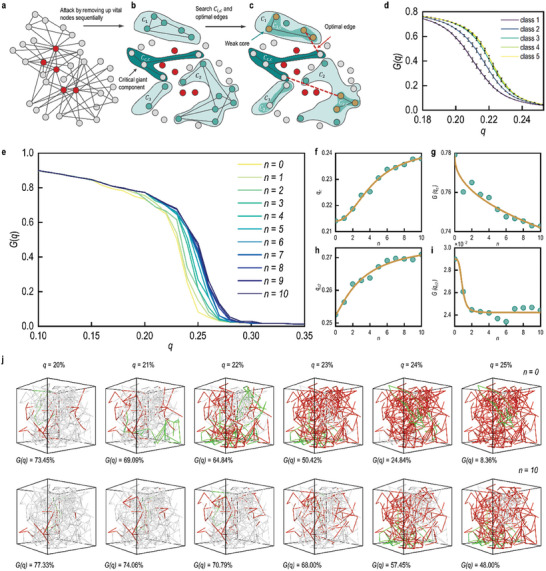
Evaluation of Structural Stability in Biomimetic Digital 3D Disordered Networks. a) Schematic diagram of the PA algorithm. The weak core of the network, comprising a proportion *q* of the total nodes, is identified based on the topological structure. b) The weak core is removed, resulting in network collapse and fragmentation into several subnetworks. The mass ratio of the largest subnetwork to the original network is denoted as *G(q)*. c) Connections are constructed between the two largest substructures to improve the network's stability against attacks.^[^ ^44^ ^]^ (insets (a–c) CC RightsLink). d) The collapse trends for each category after the networks were attacked by using the CI method. e) The evolution of the collapse curve of 3D‐DFNS under ten rounds of PA reinforcement. f) The change trend of the critical collapse point *q_c_* with each round of reinforcement. g) The change trend of the *G(q_c_)* value at the critical collapse point under ten rounds of reinforcement. h) The change trend of the critical collapse point *q_c2_* at which the largest connected substructure disappears underten rounds of reinforcement. i) The *G(q_c_)* value at the critical collapse point under ten rounds of reinforcement. j) Comparison of the collapse behavior of the original network and the network after ten rounds of PA reinforcement under the same attack index *q*. In the figure, white represents the largest substructure, green represents the second‐largest substructure, and red represents the failed parts.

Beyond the tensile stress response, a critical aspect of biological 3D‐DFNS is their structural stability when subjected to malicious attacks or local fractures. Such damage can lead to a loss of vital functions or even the collapse of the entire network. To evaluate the structural stability of biomimetic digital 3D‐DFNS, a graph‐theoretical approach was applied to evaluate the size of the largest connected component (i.e., the proportion of the largest cluster in the 3D‐DFNS) after an attack. This method quantifies stability by analyzing network integrity after an attack, using the Collective Influence (CI) method to simulate a malicious attack targeting the network. During this process, “weak nodes” were identified based on their degree attributes and subsequently removed until no significant largest cluster remained.^[^ ^43^ ^]^ To mitigate collapse, new fibers were introduced between two crosslinking points at the critical breakdown stage, reconnecting the two largest sub‐components (**Figure** **4**a).

The Posteriorly Adding (PA) algorithm was employed for 10 rounds of reinforcement on a sample of 3D‐DFNS (Figure 4b,c).^[^ ^44^ ^]^ The collapse process of biomimetic digital 3D‐DFNS is depicted in Figure 4d. For quantitative analysis, the proportion of removed nodes was used, denoted as *q*, to represent attack intensity, and the proportion of the largest connected component relative to the original mass, denoted as *G(q)*, as a measure of stability. For the five categories of 3D‐DFNS identified through clustering analysis, the changes post‐attack exhibited a three‐stage characteristic. In the early stage of the attack *q* < 19%, the proportion of connected components relative to the original mass decreased linearly with fiber removal. This trend, consistent with the reduction in total fiber length, indicated no significant collapse at this stage. However, in the mid‐stage of the attack (19% < *q* < 24%), the stability *G(q)* of 3D‐DFNS dropped sharply, signifying significant breakdown.

Given the high time complexity of the algorithm, the reinforcement was limited to 10 rounds, after which the collapse trend of 3D‐DFNS under malicious attack was significantly delayed (Figure 4e). By comparing the critical breakdown point, *q_c_*, and the largest component mass, *G(q_c_)*, before and after reinforcement, the improved stability of biomimetic digital 3D‐DFNS under malicious attack was analyzed.

The critical collapse index *q_c_* of the biomimetic biological 3D‐DFNS was determined to be 19%, significantly higher than that of non‐biological networks with high node degree or centrality, such as scale‐free networks (SF network, γ = 3, *q_c_* = 10‐15%) and power networks (q_c_ ≈ 5%). This value was comparable to that of the commonly used Erdős‐Rényi (ER)network (⟨k⟩ = 4, *q_c_* ≈ 20%), suggesting that biological 3D‐DFNS exhibit superior stability under malicious attack. This enhanced stability can be attributed to their low node crosslinking degree (primarily triple junctions) and high uniformity in crosslinking density.^[^ ^44^ ^]^

Additionally, a comparative analysis of the average collapse curves was performed for the five 3D‐DFNS categories, which were clustered based on tensile stress growth values. For one of these structures, two critical turning points were identified in the *G(q)*‐*q* graph: C1 (the attack proportion at which the 3D‐DFNS reach critical collapse) and C2 (the attack proportion at which the 3D‐DFNS complete disintegrate). We examined the changes in these critical values, *q* = *q_c_*, *q_c2_*, as well as G(*q_c_*) and G(*q_c2_*) as PA reinforcement progressed. As shown in Figure 4f–i, as the number of PA edges (*n*) increased from 0 to 10, both *q_c1_* and *q_c2_* increased, which could be fitted using a logistic function. Similarly, G(*q_c1_*) and G(*q_c2_*) decreased with increasing *n*, following a similar logistic function trend. These results suggest that as network reinforcement progresses, the network becomes increasingly resistant to collapse.

A snapshot of the 3D‐DFNS at *q* = 22% (Figure 4j) reveals substantial localized collapse, characterized by the largest cluster splitting into smaller clusters and a noticeable increase in red‐highlighted sections. In the later stage of the attack (*q* > 24%), the 3D‐DFNS disintegrated into multiple small clusters, leaving no significant large clusters. To further validate these observations in structural stability, an individual rendered 3D‐DFNS was analyzed. As shown in Figure 4j, for a specific rendered 3D‐DFNS, when *n* = 0, *G(q)* sharply decreased from 73.45% at *q* = 20% to 8.36% at *q* = 24%. In contrast, for the system with *n* = 10, *G(q)* only decreased from 77.33% to 48% within the same range of *q*, demonstrating a significant improvement in stability due to the reinforcement. This result highlights the effectiveness of reinforcement in enhancing the network's resistance to collapse under attack.

### 2.4 Prediction and Interpretability Analysis of Structure‐Property Relationships in Biomimetic Digital 3D‐DFNS

**Figure 5:**
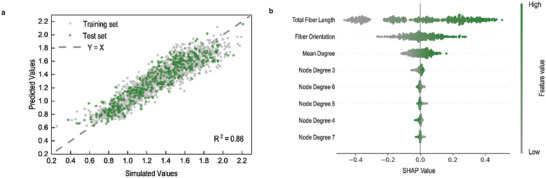
Prediction and SHAP Analysis of Structure‐Property Relationships in Biomimetic Digital 3D Disordered Networks. a) Scatter plot of simulated versus predicted stress growth values using the SVR model, which predicts stress growth values from network structure. b) SHAP summary plot for the SVR model, illustrating the impact of features on the predicted stress growth values from high to low.

By integrating graph structure analysis of 3D‐DFNS with coarse‐grained molecular dynamics simulations, a large dataset was generated that correlates 3D‐DFNS with mechanical performance. This dataset serves as the basis for predicting and optimizing structure‐property relationships in biomimetic digital 3D‐DFNS using machine learning models. Specifically, the dataset comprising 2383 sets of structure‐property correlation data, which meets the sample size requirements for common supervised learning models. Using this dataset, the algorithm of Support Vector Regression (SVR) was applied to predict stress growth values based on data of graph structure information. Additionally, SHAP values analysis was performed on the model for interpretability analysis.^[^ ^45^ ^]^ To improve the predictive accuracy of the model, the GridSearch algorithm was utilized to search for optimal hyperparameters for the SVR model. Specifically, we optimized the penalty parameter C, kernel coefficient γ, and tolerance ϵ within specified ranges, using a fixed random seed and 5‐fold cross‐validation to ensure reproducibility and robustness (Table S1, Note S1, Supporting Information). Based on the combination of these optimized hyperparameters, the SVR model achieved an R^2^ of 0.86 and a Root Mean Square Error (RMSE) of 0.13 on both the training set and test set. The consistency of these results across the training and test sets indicates that the model has been effectively tuned and generalizes well to unseen data, ensuring robust and reliable predictions of stress growth values from graph structure information. As shown in **Figure** **5**a, the scatter plot of the simulated versus predicted values for both the training set and test set indicates a close alignment of data points along the *y* = *x* dashed line, further demonstrating the robust and reliable in predicting stress growth values.

Further interpretability analysis of the features was conducted using SHAP values, as shown in Figure 5b. In this multi‐factor influence model, total fiber length and fiber orientation emerged as the most significant factors affecting the predictions of model. The average absolute SHAP value for total fiber length was 0.233, making it the most impactful feature. The wide distribution of SHAP values for total fiber length shows that higher values of this feature are strongly associated with positive SHAP values, which in turn indicates a significant increase in the predicted stress growth values. This suggests that longer fibers greatly enhance the mechanical performance of the 3D‐DFNS under stress. Fiber orientation, with an average SHAP value of 0.076, also plays an important but more moderate role compared to total fiber length. The SHAP value distribution for fiber orientation reveals a positive effect on predictions, although this influence is less pronounced. Higher fiber orientation values, which correspond to fibers aligning more closely with the tensile direction, tend to contribute positively to stress growth, but their effect is less dramatic than that of total fiber length. Mean degree, a measure of the average number of connections per node, has a smaller but still noteworthy impact. Its SHAP values are concentrated around zero, suggesting that variations in the mean degree can slightly increase or decrease the predicted stress growth values. The analysis of node degree reveals even smaller SHAP values with narrower distributions centered around zero, showing limited influence in most cases. However, there are interesting variations based on specific node degrees. For instance, Node Degree 3—which corresponds to triple junctions—shows a high density of green points on the right side of the SHAP plot, indicating that an increased number of triple junctions has a significant positive influence on stress growth predictions. Conversely, higher node degrees (e.g., Node Degree 4, 5, 6, 7) show green points on the left side of the plot, suggesting that maintaining fewer high‐degree nodes helps improved structural performance under stress. The SHAP value for nodes with degree 3 is ≈1 × 10⁻^2^, indicating a positive response in the current prediction mode. Conversely, the SHAP values for nodes with degrees 4 to 7 are ≈5 × 10⁻^3^, showing a negative response. Although higher node degrees somewhat suppress stress growth, the positive contribution of nodes with degree 3 predominates, leading to an overall positive correlation between the average degree that is primarily driven by an increase in degree 3 nodes and the model's positive predictions. These results imply that the distribution of node degree within the 3D‐DFNS affects stress growth values through different mechanisms. Specifically, an increase in triple junctions (Node Degree 3) and a reduction in high‐degree nodes contribute to enhanced mechanical performance during stretching (Figure 5b).

### 2.5 Optimization of Stability in Biomimetic Digital 3D‐DFNS

To further optimize the stability of biomimetic digital 3D‐DFNS, a Network Deep Reinforcement Learning (N‐DRL) framework was developed by integrating the malicious attack model with the Proximal Policy Optimization (PPO) algorithm. This N‐DRL framework is designed to achieve a harmonious balance between lightweight construction and exceptional mechanical properties, which are frequently encountered in the design of both natural and engineered materials. In this N‐DRL model, weight reduction serves as the primary optimization objective, while the critical collapse index (𝑞_𝑐_) is used as a secondary metric to ensure structural stability during the optimization process.

In this study, we adopt the adversarial attack model as a framework that integrates weight reduction and stability optimization. This approach enables the simultaneous optimization of both network weight and stability, achieving a balance between lightweight design and high stability. Previous research has highlighted the extensive use of adversarial attack techniques in multi‐objective reinforcement learning and network stability analysis, demonstrating superior performance in optimizing algorithm efficiency and evaluating graph structure robustness compared to traditional methods.^[^ ^44^ ^]^ Consequently, this model was applied to achieve dual optimization in 3D‐DFNS networks, focusing on weight reduction while maintaining high structural and mechanical stability. By ensuring the network retains its stability under malicious attacks, our reinforcement learning algorithm not only optimizes network performance but also enhances resilience, achieving model lightness and improving the network's safety and reliability in practical applications. This approach ensures efficient operation even in resource‐constrained environments.

**Figure 6:**
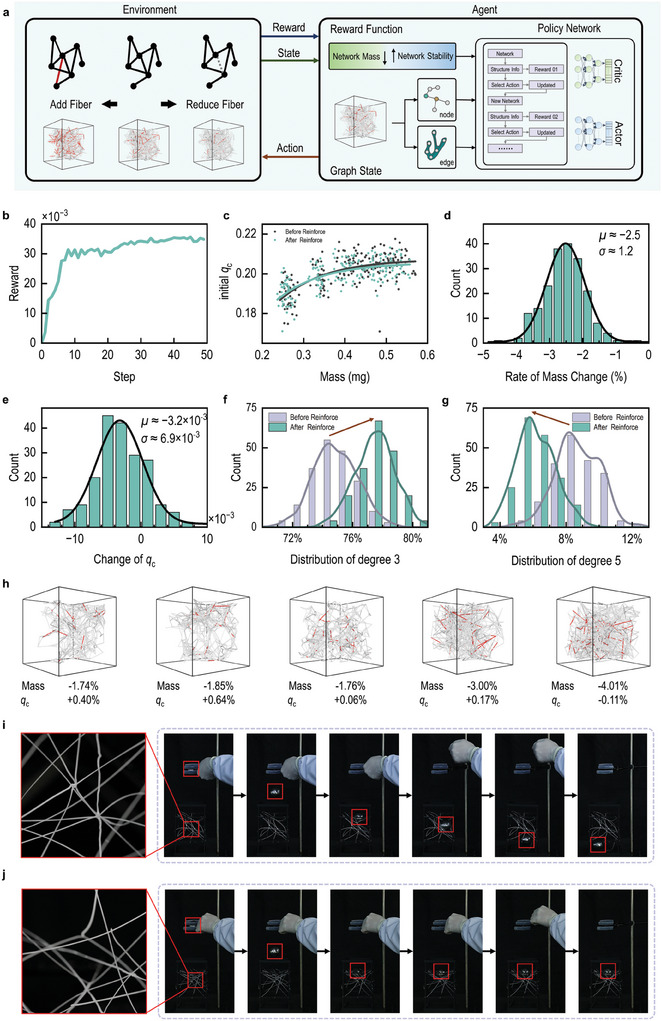
Optimization of Stability in Biomimetic Digital 3D Disordered Networks. a) Flowchart illustrating the reinforcement learning algorithm process. b) Reward increase of the N‐DRL algorithm on the test set. c) For 200 sets of 3D‐DFNS in the test set, the distribution scatter plot of the two reward components, mass and critical collapse index *q_c_* before and after reinforcement, and the corresponding trend lines. d,e) Histograms of the change in mass and the change in *q_c_* before and after N‐DRL reinforcement. f,g) Data statistical histogram of the proportion of nodes with degree 3 and the proportion of nodes with degree 5 before and after N‐DRL reinforcement. h) A comparison diagram of five 3D‐DFNS before and after N‐DRL reinforcement. The red parts indicate the fibers that were removed. i) The pre‐reinforcement 3D‐DFNS is penetrated by a 40 g ball with a diameter of 4.5 cm dropped from a height of 15 cm. j) The post‐reinforcement 3D‐DFNS successfully captures the same ball under identical conditions, maintaining structural stability without damage.

The architecture of the N‐DRL model consists of two key components: the policy network (Actor) and the value network (Critic) (**Figure** **6**a). The policy network is responsible for generating potential operations, such as adding or removing fibers between nodes with specific degree values. Meanwhile, the value network evaluates the current state of 3D‐DFNS, considering its performance under malicious attack model, and provides feedback to the policy network for decision‐making.

The N‐DRL model operates as follows: First, it receives an initial 3D‐DFNS and parses its structural features. The policy network then analyzes the data and selects the optimal action strategy. After implementing the selected operation, the 3D‐DFNS is updated accordingly, the value network evaluates the value of updated 3D‐DFNS, which is then fed back to the policy network for iterative optimization. The model is trained using the RMSprop optimizer with a learning rate of 5×10^−5^, ensuring both computational efficiency and structural stability throughout the optimization process.

2000 3D‐DFNS was randomly selected for training and 200 3D‐DFNS was reserved for inference testing. After training, the model demonstrated excellent performance on the test set, as evidenced by the significant increase in reward values, as shown in Figure 6b. The relationship between mass and the critical collapse index remained consistent before and after reinforcement, with the model showing superior performance by achieving lower mass values at the same *q_c_* (Figure 6c). On average, the network mass was reduced by 2.51% ± 0.02% after reinforcement, while the critical collapse index *q_c_* decreased by an average of 0.0032 ± 0.0003 (Figure 6d,e). Since the network remains in the early stages of attack during critical collapse, where *G(q)* decreases linearly, the N‐DRL model successfully achieved its goal of reducing weight effectively while maintaining structural stability.

The geometric features of the network were also used to analyze the topological changes in network structure during the reinforcement process. As shown in Figure 6f, the proportion of nodes with a degree of 3 increased significantly as inference rounds progressed, rising from 74.85% before reinforcement to ≈77.48%, with an average increase of 2.63%. Conversely, the number of high‐degree nodes (e.g., nodes with a degree of 5) significantly decreased from 8.68% to ≈6.17%, with an average reduction of 2.51% (Figure 6g).

Notably, the N‐DRL model demonstrated significantly faster inference speeds compared to traditional heuristic algorithms such as the PA algorithm.^[^ ^44^ ^]^ For instance, for networks with the same structure, the N‐DRL model completed inference in ≈1 minute per round, with the PA method took ≈120 minutes per round (Figure 1e). This speed advantage is crucial for optimizing large numbers of biomimetic 3D‐DFNS, where time cost is a key consideration.

Furthermore, the performance of N‐DRL on 3D‐DFNS across different mass levels was evaluated. In low‐mass configurations, N‐DRL enhances stability while achieving a slight reduction in mass. Conversely, for high‐mass setups, it significantly decreases mass without compromising stability (Figure 6h).

By comparing snapshots of the networks before and after reinforcement, the fiber length was significantly increased after reinforcement. This structural change is similar to the phenomena observed in nature, such as the use of long‐span radial threads in spider webs to maintain the overall structure, as well as the distribution of a large number of shorter edges and a smaller number of longer edges within the silk microfibrous networks. For example, in silk microfibrous networks, longer fibers dominate, with most nodes exhibiting a degree of 3, leading to exponential growth in axial stress.^[^ ^46^ ^]^ In contrast, nonwoven networks or diseased cytoskeleton networks tend to have shorter, more uniform fibers and a higher density of high‐degree nodes, resulting in stable but less variable moduli.^[^ ^47^, ^48^ ^]^

To validate the effectiveness of our N‐DRL model in optimizing 3D‐DFNS, 3D printing was employed to fabricate two 3D‐DFNS architectures with consistent mass, based on geometric configurations directly extracted from the designs before and after reinforcement learning optimization. The structural stability of these architectures was tested through a drop ball impact test. As shown in Figure 6i,j and Movie S1 (Supporting Information), the pre‐reinforcement 3D‐DFNS (Figure 6i) was penetrated by the falling ball. In contrast, the post‐reinforcement 3D‐DFNS (Figure 6j) captured the ball, with the network structure remaining stable and undamaged. These results confirmed that the N‐DRL model we developed is effective for optimizing the network materials for practical applications.

Thus, the N‐DRL model not only optimized the mechanical tunability of the network but also provided a general strategy for enhancing lightweight, high‐strength network materials. This strategy involves increasing the number of triple junctions, reducing high‐degree crosslinking points, and incorporating connections longer than the average fiber length.

## 3 Conclusion

This study presents a comprehensive investigation into biomimetic 3D‐DFNS, revealing key insights into their structure‐property relationships and stability. By combining procedural modeling, coarse‐grained molecular dynamics simulations, machine learning, the complex mechanical behaviors of these networks were successfully analyzed. The N‐DRL framework developed in this work further enabled the optimization of network stability, balancing the weight reduction and structural integrity. Our findings indicate that total fiber length is a critical factor influencing mechanical performance, while the influence of fiber orientation is more moderate. Networks composed of longer fibers exhibited enhanced stability due to more effective stress dispersion. Furthermore, the structural integrity of the networks was improved by increasing the number of triple junctions and reducing the presence of high‐degree nodes. These structural modifications contributed to a more efficient and robust network architecture. The N‐DRL model outperformed traditional heuristic methods, providing a more efficient and scalable approach to optimizing these networks in terms of both mass reduction and computational time. Overall, this research bridges a significant gap in the application of 3D‐DFNS to engineered materials. It provides a novel framework that integrates machine learning and N‐DRL to optimize mechanical tunability and structural stability of biomimetic 3D‐DFNS. The strategy developed here offer valuable guidelines for designing lightweight, high‐strength materials, applicable to both natural and engineered systems.

## 4 Experimental Section

### Artificial Network Construction

To construct a biomimetic network, pixels within a cube with a side length of 1000 pixels were randomly selected as nodes, represented by the vector (n_i1_, n_i2_, n_i3_). If the distance between this node and all previously generated nodes is greater than the preset minimum connection distance *l_min_*, this node was accepted as *n_i_*. This process continues until the number of nodes reaches the preset upper limit *n_max_*, or multiple consecutive random selections are rejected, thereby stopping the generation of nodes. Next, all node pairs {*n_i_*, *n_j_*} were traversed to determine whether a connection should be formed between each pair of nodes. Several constraints were applied to ensure that the random network was statistically similar to a specific 3D spider web in terms of segment length and node connectivity. The first constraint was the connection length, requiring that the maximum connection length satisfy *l_ij_* ≤ *l_max_*. Node pair {*n_i_*, *n_j_*} meeting this condition were added to the neighbor list. The constraint on the number of node connections was also imposed. For each node, a node degree *d_r_* is randomly assigned based on the degree distribution reported in published studies.^[^ ^2^ ^]^ If a node is already connected to other nodes with a degree of *d_0_* and *d_r_* > *d_0_*, the node is then connected to *d* = *d_r –_ d_0_* nearest nodes. If *d_r_* ≤ *d_0_*, no additional connections are established.

### Verification and Cleaning of the Network

Once the fiber network is obtained, all free‐end nodes with a connection number *d_i_* = 1 were recursively removed. Subsequently, a connectivity analysis was performed on the entire structure, with only the largest connected branch being retained. This process ensures that a coherent and well‐connected structure was obtained.

### Bead‐and‐Stick Model

By setting an actual length *l_real_* = 0.1 m as the side length of the simulation box, the vector of each node in the model space was converted into coordinates (*x_i_, y_i_, z_i_*) in real space, utilizing the International System of Units (SI) for modeling. Each pair of connected nodes was treated as forming a fiber, with constraints imposed on the fiber length distribution. Fiber lengths were calculated based on the node coordinates, and all fibers were sorted by length. The longest and shortest fibers were kept fixed, while the remaining fiber lengths are remapped according to the distribution shown in Figure 1d. According to the reported density *ρ* = 1.3 g/cm^3^ and fiber diameter *d* = 4.34 µm of the *Cyrtophora citricola* spider web,^[^ ^40^ ^]^ the mass *m_i_* of a single node *i* is defined as half the total mass of all fibers connected to that node.

(2) $$m_{i}=\frac{\pi d^{2}\rho }{8}\sum_{J}l_{i,j}$$

where *J* is the set of nodes *j* connected to node *i* and *l_i,j_* is the fiber length between node *i* and node *j*. Each fiber was considered it as a linearly elastic body, and the harmonic potential energy of the bond interaction is defined by equation (1). The fiber mass is concentrated at the nodes, with the mass of each crosslinking point being half the total mass of all connected fibers. In addition, a Lennard‐Jones potential was incorporated, with both the equilibrium separation and the cutoff distance set to the fiber diameter, to prevent particle penetration. The formula for the Lennard‐Jones potential is:

(3) $$U_{\mathrm{lJ}}=4\varepsilon \left[{\left(\sigma r\right)}^{12}-{\left(\sigma r\right)}^{6}\right],r<r_{c}$$

where *ε* is the potential well, σ is the zero‐potential point, and r_c_ is the cutoff distance.

### Stress Simulation by Molecular Dynamics

The obtained fiber network model is pre‐equilibrated under a 10 K microcanonical ensemble (NVE) and then output as the initial structure for 3D‐DFNS. Then, the leftmost and rightmost 5% of the atoms along the x‐axis are fixed, and a stretching simulation is performed. This is achieved by iteratively deforming the simulation box and performing energy minimization at each increment using the Polak‐Ribiere conjugate gradient algorithm. For each fiber network, a corresponding stress‐strain curve is obtained.

### Extraction of Graph Structural Information in 3D‐DFNS

The extraction of the graph structure in 3D‐DFNS is primarily conducted using Python (version 3.8) and NetworkX (version 3.1). After reading the 3D‐DFNS data stored in NetworkX JSON format, the program leverages NetworkX's API to extract features such as Total Length, Mean Degree, and Node Degrees ranging from 3 to 7. The Fiber orientation feature is calculated by representing the direction of each fiber as a unit vector and quantifying its alignment relative to the stretching direction (x‐axis). Structural information from 2383 instances of 3D‐DFNS is extracted and combined with the corresponding stress growth values to form the dataset. The Pearson Correlation Coefficient was utilized to measure the linear correlation between variables. The Pearson correlation coefficient is defined as:

(4) $$\begin{array}{c} \;\rho_{X,Y}=\frac{\mathrm{Cov}\left(X,Y\right)}{\sigma_{X}\sigma_{Y}}=\frac{\sum_{i=1}^{n}\left(X_{i}-\bar{X}\right)\left(Y_{i}-\bar{Y}\right)}{\sqrt{\sum_{i=1}^{n}{\left(X_{i}-\bar{X}\right)}^{2}}\sqrt{\sum_{i=1}^{n}{\left(Y_{i}-\bar{Y}\right)}^{2}}}\; \end{array}$$

where Cov(*X*, *Y*) denotes the covariance of variables *X* and *Y*, σ_*X*_ and σ_*Y*_ are the standard deviations of *X* and *Y* respectively, *n* is the sample size, $\bar{X}$ and $\bar{Y}$ represent the sample means of *X* and *Y*, respectively.

### Machine Learning for Predicting Stress Growth Values in 3D‐DFNS and SHAP analysis

A Support Vector Regression (SVR) model was developed using Python software (version 3.8) and scikit‐learn library (version 1.3.2) to accurately predict stress growth values from the fiber network structure in 3D‐DFNS. The dataset was randomly divided into training and test sets, with the test set comprising 20% of the total data. Subsequently, feature standardization was performed to ensure that the features of both the training and test sets followed a standard normal distribution, with a mean of 0 and a standard deviation of 1. Hyperparameters of the SVR were then optimized using a combination of Grid Search and Cross‐Validation. The hyperparameter combination corresponding to the model with the lowest RMSE score was selected as the hyperparameter of the model. Finally, the model was evaluated on the test set using RMSE and the coefficient of determination (R^2^) as evaluation metrics to obtain the prediction accuracy of model for unseen data. The explainability analysis of the SVR model was conducted using SHAP library (version 0.44.1) in Python. Shapley values, grounded in cooperative game theory, were utilized to allocate each feature's contribution to the prediction outcome. For a given prediction function *f* and feature set F, the Shapley value for the *i*‐th feature is defined as:

(5) $$\phi_{i}\left(f\right)=\sum_{S\subseteq F\setminus \left\{i\right\}}\frac{\left|S\right|!\left(\left|F\right|-\left|S\right|-1\right)!}{\left|F\right|!}\left[f\left(S\cup \{i\}\right)-f\left(S\right)\right]$$

where *S* is a subset of features, *f*(*S*) represents the model output when only the feature subset *S* is used for prediction, |*S*| is the number of features in subset *S*, and |F| is the total number of features in the feature set F.

### Enhancement of 3D‐DFNS

The N‐DRL framework is implemented using Python (version 3.8) and PyTorch (version 2.0.1+cu118), and operates on a workstation equipped with an Intel i7‐14700k CPU and an RTX 4090D GPU. This framework integrates an adversarial attack model with the Proximal Policy Optimization (PPO) algorithm, aiming to achieve the dual optimization goals of weight reduction and high structural stability in 3D‐DFNS. The N‐DRL model comprises an Actor network and a Critic network. The Actor network is responsible for generating actions such as adding or removing fibers between nodes of specific degrees, while the Critic network evaluates the current structural state and provides feedback to the policy network to guide decision‐making. During the training process, 2000 instances of 3D‐DFNS were used for training and 200 instances were reserved for testing. Feature standardization is applied using the RMSprop optimizer with a learning rate set to 5 × 10^−5^. The PPO objective function optimizes the policy by maximizing cumulative rewards, and it is mathematically expressed as:

(6) $$L^{\mathrm{CLIP}}\left(\theta \right)=E_{t}\left[\min\left(r_{t}\left(\theta \right)\hat{A_{t}},\mathrm{clip}\left(r_{t}\left(\theta \right),\;1-\in ,\;1+\in \right)\hat{A_{t}}\right)\right]$$

(7) $$\begin{array}{c} r_{t}\;\left(\theta \right)=\frac{\pi_{\theta }\left(a_{t}|s_{t}\right)}{\pi_{\theta_{\mathrm{old}}}\left(a_{t}|s_{t}\right)}\; \end{array}$$

where *L* ^CLIP^(θ) is the PPO objective function, *E_t_* denotes the expectation over time step *t*, *r_t_* (θ) is the policy ratio, $\hat{A_{t}}$ represents the advantage function, ε is the clipping parameter (set to 0.2), *s_t_* indicates the state at time step *t* (typically referring to the current structural features of the 3D‐DFNS), and *a_t_* represents the action taken in state *s_t_*, such as adding or removing fibers between nodes of specific degrees. This objective function ensures stable learning by clipping the policy update step size, thereby preventing excessive deviation and maintaining the stability of the learning process. The model is configured to terminate operations upon reaching a specified number of iterations and achieving the dual optimization objectives.

## Conflict of Interest

The authors declare no conflict of interest.

## Supporting information



Supporting Information

Supplemental Movie 1

## Data Availability

The data that support the findings of this study are available from the corresponding author upon reasonable request.
