# Supplementary material for: Optimizing Biomimetic 3D Disordered Fibrous Network Structures for Lightweight, High‐Strength Materials via Deep Reinforcement Learning
Source: Adv Sci (Weinh). 2025 Jan 23;12(11):2413293. doi: 10.1002/advs.202413293 (PMC11923916; doi:10.1002/advs.202413293)
Supplement: Supplementary file 1 — Supporting Information [file ADVS-12-2413293-s002.docx]

Supplementary Information

**Optimizing Biomimetic Three-Dimensional Disordered Fibrous Network Structures for Lightweight, High-Strength Materials via Deep Reinforcement Learning**

*Yunhao Yang#, Runnan Bai#, Wenli Gao*, Leitao Cao, Jing Ren, Zhengzhong Shao, and Shengjie Ling**

**Supplementary information contains:**

Table S1

Note S1

**Table S1.** Hyperparameter Optimization for SVR.

| Hyperparameter | Description | Search Range |
| --- | --- | --- |
| C | Penalty parameter | 10^2^ - 10^4^ |
| γ | Kernel coefficient | 10^-4^ - 10^2^ |
| ε | Tolerance for stopping criteria | 5*10^-2^ – 2*10^-1^ |

**Note S1.** Hyperparameter Optimization for SVR: Following the hyperparameter tuning, the Support Vector Regression (SVR) model was configured with the optimal parameters: C = 100.0 ,γ = 0.001, and ϵ = 0.1. The dataset was divided into training and testing sets using an 80-20 split with a fixed random seed 42 to ensure reproducibility. Prior to training, feature scaling was performed using StandardScaler to normalize the input variables. A 5-fold cross-validation strategy was employed during the training process to enhance the robustness and reliability of the model.
